# Identification and Validation of Novel Lipids Linked to Bone Mineral Density Change and Fracture Risk

**DOI:** 10.1007/s00223-025-01399-1

**Published:** 2025-06-25

**Authors:** Canchen Ma, Ziyuan Shen, Jing Tian, Yvette L. Schooneveldt, Corey Giles, Flavia Cicuttini, Graeme Jones, Peter J. Meikle, Feng Pan

**Affiliations:** 1 Menzies Institute for Medical Research, University of Tasmania, Private Bag 23, Hobart, TAS 7000 Australia; 2 Department of Rheumatology, School of Medicine, Renji Hospital, Shanghai Jiaotong University, Shanghai, China; 3 Department of Epidemiology and Biostatistics, School of Public Health, Anhui Medical University, Hefei, 230032 Anhui China; 4 Baker Heart and Diabetes Institute, Melbourne, Australia; 5 Department of Cardiovascular Research, Translation and Implementation, La Trobe University, Bundoora, VIC 3086 Australia; 6 Department of Epidemiology and Preventive Medicine, Monash University Medical School, Commercial Road, Melbourne, Australia

**Keywords:** Bone mineral density, Fracture, Lipids, Mendelian randomization

## Abstract

To identify and validate lipid metabolites associated with bone mineral density (BMD) change and fracture risk through integrated Mendelian randomization (MR) and observational analyses. Two-sample MR analysis was first performed to uncover potential causal relationships between 32 lipid classes and 576 lipid species and BMD and fractures. Identified signatures were subsequently validated in an independent cohort (N = 492), where lipids, BMD, and fracture status were measured at two time points, 8 years apart. The false discovery rate method was employed to control multiple testing. Linear and log binomial mixed-effects models were used to analyze lipid associations with hip BMD and fracture risk, respectively. Two-sample MR revealed seven lipid classes causally associated with BMD and/or fractures, including acylcarnitine (AC), cholesteryl ester (CE), sphingomyelin (SM), phosphatidylinositol (PI), GM3 ganglioside (GM3), alkylphosphatidylcholine (PC(O)) and triacylglycerol (TG). Causal associations were found between 18 lipid species across these classes and BMD, and 10 lipid species were associated with fractures. Validation in an independent longitudinal cohort confirmed associations for total SM, SM(d18:1/16:0), SM(d18:2/24:0), and CE(18:3) with hip BMD change (β ranging from − 0.036 to − 0.012 g/cm^2^, per log µM increase, p < 1.13 × 10^–2^). Total SM, total GM3, and SM(d18:2/18:1), SM(d18:2/22:0), SM(d18:2/17:0) were associated with an increased risk of fractures (RR ranging from 1.038 to 1.290 g/cm^2^, per log µM increase, p < 5 × 10^–2^) over 8 years. Our findings suggest that alterations in lipid metabolism play a causal role in bone remodeling and fracture risk. This warrants further investigation into the mechanisms of lipid-mediated BMD changes and the potential for identifying patients at ‘high risk’ of osteoporotic fracture.

**Graphical abstract:**

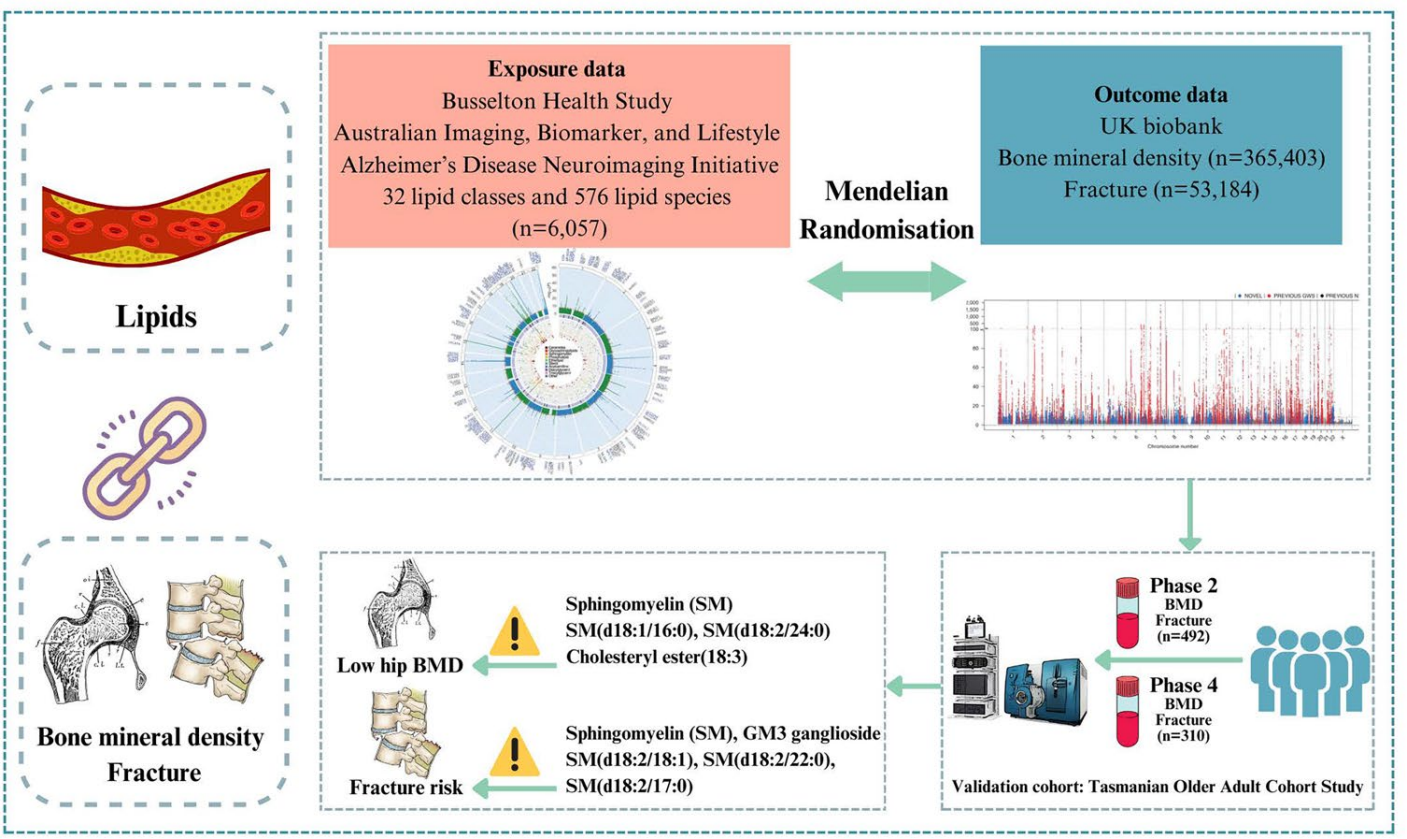

**Supplementary Information:**

The online version contains supplementary material available at 10.1007/s00223-025-01399-1.

## Introduction

Osteoporotic fractures present a major health concern, particularly in the elderly, with low bone mineral density (BMD) as a strong risk factor [1]. Osteoporotic fractures affect approximately one in two women and one in five men over the age of 50 [2], which have a high impact on the health-related quality of life of patients. The identification of modifiable risk factors and biomarkers associated with low BMD and fractures is crucial for developing new preventive and treatment strategies, ultimately leading to a reduction in morbidity, mortality, and healthcare costs [3].

Although several population-level studies have reported associations between circulating biomarkers (e.g., osteocalcin and C-Terminal Telopeptide of Type I Collagen) and bone metabolism and fracture risk, their predictive accuracy and clinical utility remain limited and inconsistent [4, 5]. Lipids, as a basic component of cell membranes, together with proteins and nuclei acids, build biological membranes and cells and play vital roles as signaling molecules and energy stores in medical conditions, as well as in bone health [6]. Recent observational studies have explored the association of clinical lipid profiles with BMD and fractures; however, these studies are observational in nature and cannot establish causality [7, 8]. For example, elevated levels of low-density lipoprotein (LDL) have been found to be associated with incident fractures, while lower levels of high-density lipoprotein (HDL) have been correlated with BMD loss in lumbar and femoral regions in postmenopausal women [9, 10].

Mendelian randomization (MR) uses genetic variants as instrumental variables (IVs) to estimate causal effects and reflects the underlying genetic predispositions that influence modifiable exposures and health outcomes [11]. Studies using MR have reported that LDL and HDL alterations may be causally associated with osteoporosis risk [12, 13]. However, LDL and HDL are composite measures representing the cholesterol content of large, complex lipoprotein particles. These lipoproteins are made up of thousands of individual lipid species that each have unique biological roles. The roles of these individual lipid species and their effects on bone health remain to be elucidated. Additionally, prior MR studies were constrained by relatively small sample sizes [14, 15].

Lipidomics technology can measure hundreds of individual molecular lipids that make up the human lipid profile, providing a more comprehensive and detailed understanding of lipid metabolism than traditional lipid measurements [16]. This advanced method may not only further facilitate our understanding of the role of lipids in bone disorders, but also help identify new therapeutic targets. However, to date, a comprehensive investigation of lipid metabolites for BMD changes and fracture risk has not been evaluated. Therefore, this study was to identify and validate lipid metabolites associated with changes in BMD and fracture risk by integrating evidence from MR and observational analyses.

## Materials and methods

### Mendelian randomization analysis of lipids for BMD and fracture risk

#### Data sources

**Fig. 1:**
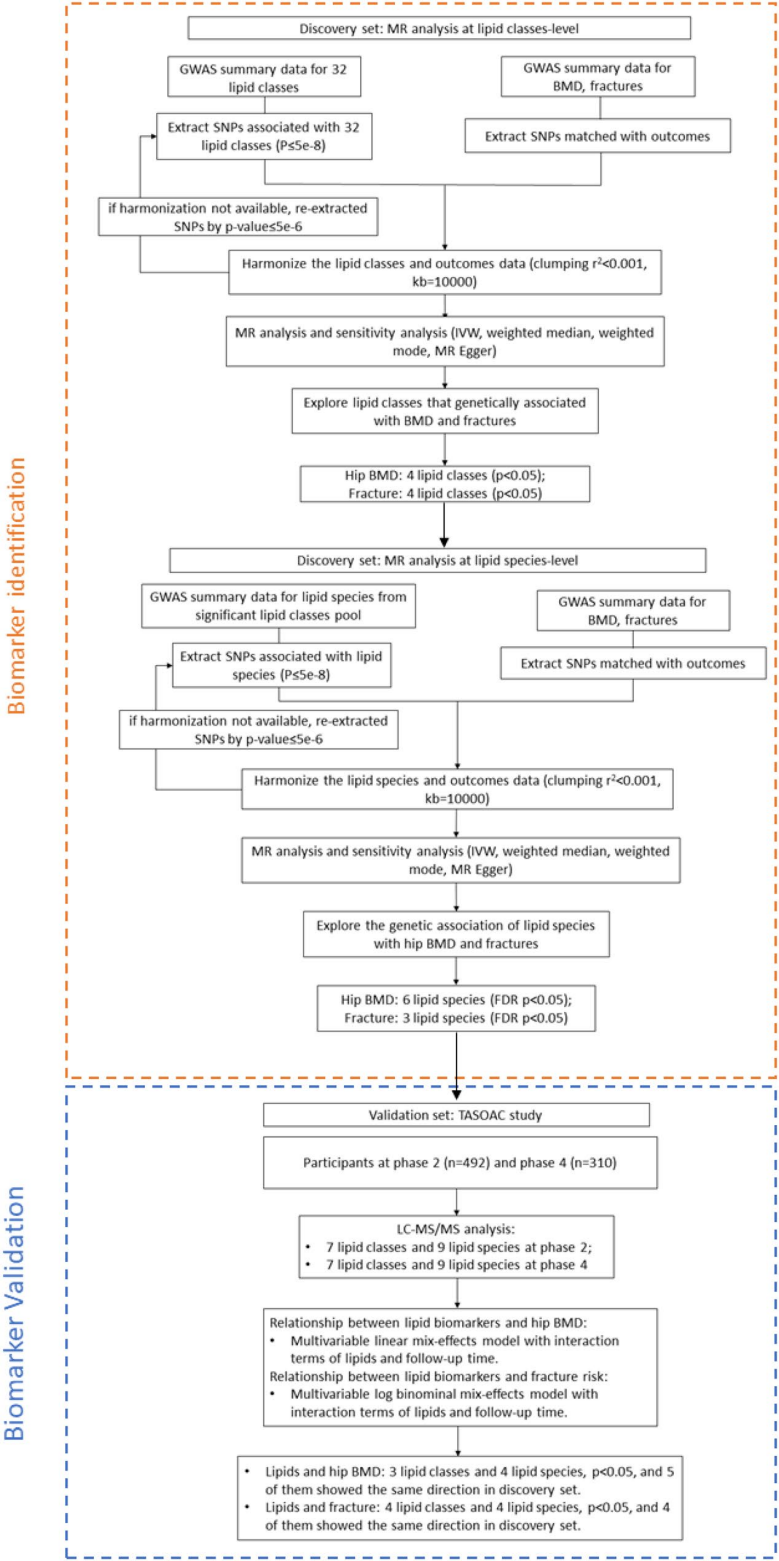
Flowchart of this study design. MR: Mendelian Randomization; BMD: Bone Mineral Density; GWAS: Genome-Wide Association Study; SNP: Single Nucleotide Polymorphism; IVW: Inverse-Variance Weighted; FDR: False Discovery Rate; TASOAC: Tasmanian Older Adult Cohort; LC–MS/MS: Liquid Chromatography–Tandem Mass Spectrometry

Single nucleotide polymorphisms (SNP) related to the human lipidome were selected as IVs from our previous genome-wide association study (GWAS) (Fig. 1 and Graphical abstract) [16]. The study was a large-scale GWAS of the human serum lipidome data from 6,057 participants (individuals of European ancestry). The summary-level data for heel BMD from the UK biobank were selected (IEU Open GWAS Project ID: ebi-a-GCST90014022), which included 365,403 individuals of British ancestry [17]. Heel BMD was assessed using quantitative ultrasound (Hologic Sahara system) in the UK Biobank, with standardized T-scores derived from speed of sound and broadband ultrasound attenuation. The summary-level data for osteoporotic fracture was obtained from the UK Biobank (GWAS Catalog ID: GCST006980), which included 53,184 British ancestry cases and 373,611 British ancestry controls [18]. Osteoporotic fracture cases were identified through both hospital records using International Classification of Diseases (ICD)-10 codes (excluding traumatic/pathological fractures) and questionnaire-based self-reports of fractures within the past 5 years. There was no overlap between the populations used for exposure (lipidome) and outcomes (BMD and fracture).

#### Instrumental variable selection

SNPs associated with lipid class/species at the genome-wide significance threshold (p < 5 × 10^–8^) were selected as potential IVs. The reference panel for assessing linkage disequilibrium (LD) between these SNPs consisted of the European contingent from the 1000 Genomes Project. The LD threshold for clumping was established at r^2^ < 0.001, with a clumping window size set to 10,000 kb. SNPs with minor allele frequency (MAF) ≤ 0.01 were excluded, along with palindromic A/T or G/C alleles. If harmonization was not available, or the number of extracted SNPs was fewer than 3, then IVs were re-extracted using a threshold of p < 5 × 10^–6^.

### Observational analysis in Tasmanian Older Adult Cohort (TASOAC) study

#### Study population

The TASOAC Study, a prospective population-based cohort study, recruited 1,099 individuals aged 50 to 80 years at baseline, who were selected randomly from the electoral roll in Southern Tasmania [19]. Participants then underwent follow-up assessments at 2.6 years (phase 2), 5.1 years (phase 3), and 10.7 years (phase 4). The number of attendees at each follow-up was 875, 768, and 566, respectively. Blood samples were obtained at the 2.6- and 10.7-year follow-up and used for the lipidomics assay. This study comprised 492 and 310 participants with complete data on BMD, fracture, lipidomics, and covariates at the 2.6- and 10.7-year follow-ups (Fig. 1 and Graphical abstract). Approval for the TASOAC study was granted by the Southern Tasmanian Health and Medical Human Research Ethics Committee (Ref. no: H0006488), and written informed consent was obtained from all participants.

#### Lipidomics profiling

Lipidomics profiling was measured at both 2.6- and 10.7-year follow-ups. Blood samples were obtained following a minimum 8-h fasting period and subjected to centrifugation at 13,000 xg for 10 min. This fasting duration aligns with standard protocols for lipidomic profiling and is considered sufficient to reduce postprandial variation in circulating lipid levels, as validated in previous studies using liquid chromatography tandem-mass spectrometry (LC–MS/MS) [20, 21]. Serum samples were randomized, and lipid extraction was performed using the butanol/methanol method, as described previously [22]. Lipidomic analysis was conducted using an Agilent 6495C triple quadrupole LC-MS/MS in conjunction with an Agilent 1290 series HPLC system and a ZORBAX eclipse plus C18 column (Agilent, Santa Clara, CA, USA) operating in positive/negative switching polarity, as detailed previously [22, 23]. More details were given in Supplementary file 1.

#### Hip BMD

The dual-energy X-ray absorptiometry (DXA) (Hologic Delphi densitometer, MA, USA) was employed to conduct the total hip BMD scans at both 2.6- and 10.7-year follow-ups.

#### Fractures

Fractures were self-reported and documented at both 2.6- and 10.7-year follow-ups. Participants were asked: “List any fracture you may have had since your previous interview for this study. Please list these by the location of the fractures (including vertebral, non-vertebral, hip and major fractures involving the femur, radius, ulnar, vertebral, rib and humerus).”

#### Covariate measurements

Age, sex, and current smoking status were collected through a questionnaire during an interview at 2.6 years. Standing height and body weight were measured, and body mass index (BMI, kg/m^2^) was calculated. Physical activity was measured by recording steps per day over seven consecutive days utilizing a pedometer (Omron Healthcare, Kyoto, Japan) at 2.6 years. The detailed criteria for inclusion of pedometer estimates have been described previously [24]. Serum 25-hydroxyvitamin D (25(OH)D) concentrations were measured using liquid-phase radioimmunoassay (Immunodiagnostics Systems Ltd), which detects both 25(OH)D_2_ and 25(OH)D_3_. The intra-assay and inter-assay coefficients of variation were 1.8% and 3.3%, respectively [25]. Falls risk scores were derived from the physical profile assessment (PPA) at 2.6 years. The PPA is a validated and reliable tool that assesses five physiological domains: visual contrast sensitivity, reaction time, knee extension strength, proprioception, and postural sway on a foam surface, and standardized Z-scores based upon these five domains, were then calculated [26]. Statin use was self-reported at the 2.6-year follow-up. Energy-adjusted dietary inflammatory index scores were calculated based on validated food frequency questionnaire data collected at baseline, as previously described [27].

### Statistical analysis

#### Two-sample MR and sensitivity analyses

In the two-sample MR analysis, the estimation of the causal effect of exposure on the outcome was carried out using the inverse-variance weighting (IVW) method. This involved combining the ratio estimates for each SNP and effectively translating MR estimates into a weighted regression of SNP-outcome effects on SNP-exposure effects [28]. The fixed-effect IVW was used as the primary analysis method. In cases where there was significant heterogeneity among the IVs, the multiplicative random effect inverse-variance weighted (IVW-MRE) method was utilized to analyze associations. MR-Egger serves as a tool for estimating causal effects through the slope coefficient from Egger regression, and it also can detect small study bias and certain forms of pleiotropy [29]. The weighted median method offers unbiased estimates, even when up to 50% of the information is derived from invalid IVs [30]. Furthermore, the weighted mode approach assumes that the most frequently occurring association estimate remains unaffected by pleiotropy, indicating that it corresponds to the true causal effect [31].

Cochran’s Q statistic, MR-PRESSO analysis, F-statistic and Steiger filtering test method were described in Supplementary file 1.

For all lipid classes, we initially performed MR analysis at the lipid classes-level to explore their associations with BMD/fracture. Subsequently, within the identified significant lipid classes, further MR analyses were performed at lipid species-level, by examining their relationships with the outcomes. The false discovery rate (FDR) correction was used to control multiple testing for analyses at the lipid species-level.

The two-sample MR analyses were performed using the TwoSampleMR (version 0.5.8), Mendelian randomization (version 0.8.0), and MR-PRESSO (version 1.0) in R Software 4.3.2 (https://www.R-project.org). FDR-corrected p-value < 5 × 10^–2^ was considered statistically significant after controlling multiple testing.

#### Descriptive analysis in the validation cohort

Participants' characteristics were described using the mean (standard deviation [SD]) for continuous variables and the percentage (number) for categorical variables. The concentrations of lipid classes and species were natural log transformed for normalization and used for analyses.

#### Associations between lipids and hip BMD over 8 years

Linear mixed-effects models were used to analyze the associations between classes and species of lipids and hip BMD measured at both the phase 2 and phase 4, after adjusting for age, sex, BMI, physical activity, current smoking status, serum levels of vitamin D at phase 2. The interaction terms of lipids and follow-up time in the same linear mixed-effects models were used to estimate the associations between lipid classes and lipid species and changes in hip BMD over 8 years.

#### Associations between lipids and fracture risk over 8 years

Log binominal mixed-effects models were used to analyze the associations between classes and species of lipids and fracture risk, which were recorded at both the phase 2 and phase 4. These models were adjusted for age, sex, BMI, physical activity, current smoking status, falls risk score, and time-dependent hip BMD. The interaction terms of lipids and follow-up time in the same log binomial mixed-effects models were used to estimate the associations between classes and species of lipids and fracture risk over 8 years.

#### Sensitivity analyses

Sensitivity analyses were performed by adjusting for statin use and dietary inflammatory index at the 2.6-year follow-up. Additionally, inverse probability weighting method was applied to account for potential attrition bias.

Stata software (V.17) (Stata Corp, College Station, TX, USA) was used to conduct the observational analyses. Statistical significance was defined as a two-tailed p-value < 5 × 10^–2^.

## Results

### Biomarker identification

There were 500 SNPs associated with lipid classes at a significance level of p < 5 × 10^–6^ and selected as IVs (Supplementary file 2, Table S1). The F-statistics for the IVs ranged from 18.90 to 611.91, indicating no evidence of weak instrument bias (Supplementary file 2, Table S2-S3). The results of the Steiger filtering analysis indicated that all the associations identified by MR (including MR sensitivity analyses) had the correct causal directions from the lipid classes and species to the BMD and fracture (data not shown).

### MR analyses of lipid classes for BMD and fracture

**Fig. 2:**
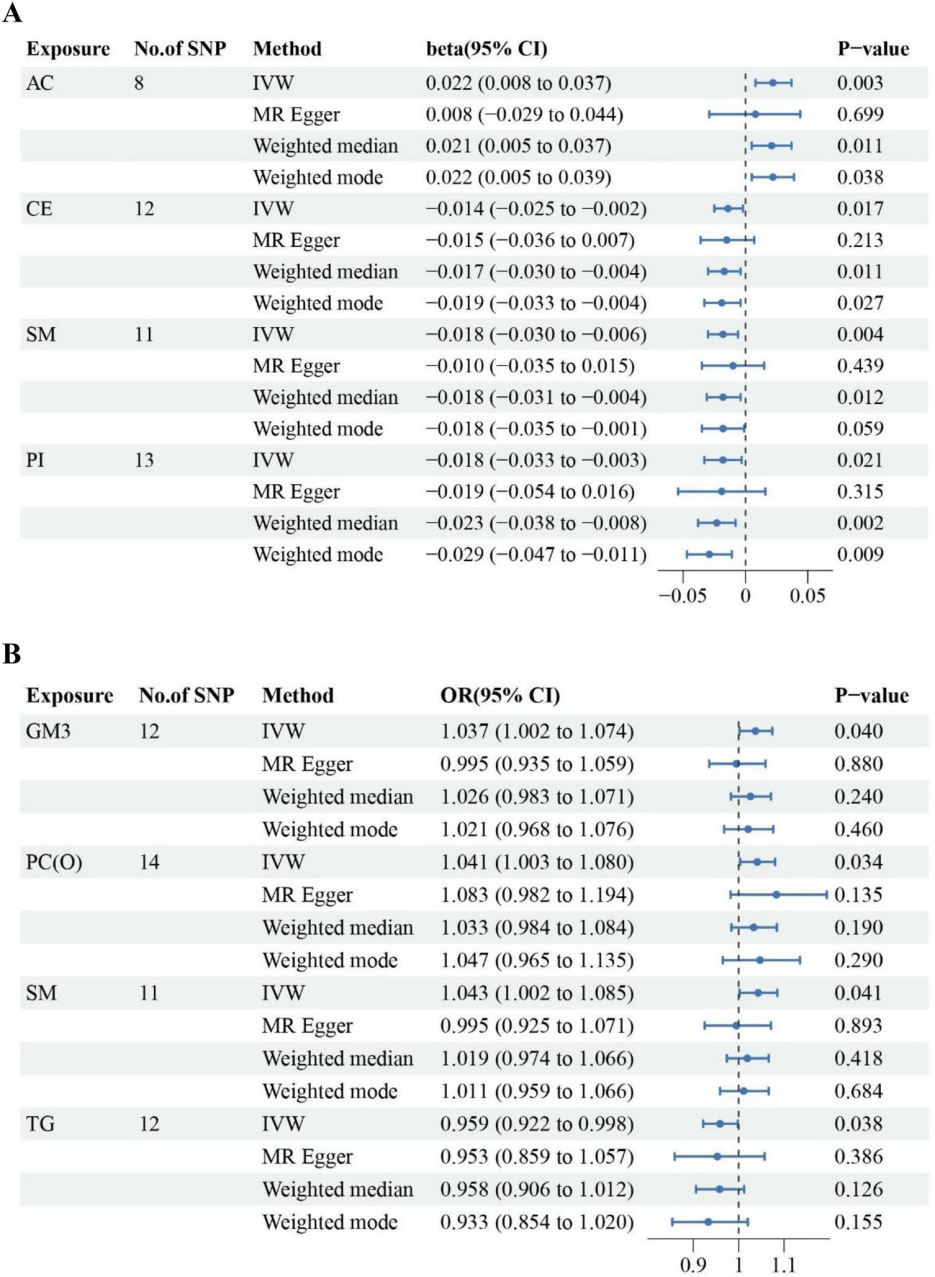
MR analyses at lipid class-level. **A**. Forest plot of MR estimates for the association between lipid classes and bone mineral density. **B**. Forest plot of MR estimates for associations between lipid classes and fracture risk. SNP: Single Nucleotide Polymorphism; CI: Confidence Interval; OR: Odds Ratio; IVW: Inverse-Variance Weighted; AC: Acylcarnitine; SM: Sphingomyelin; CE: Cholesteryl ester; PI: Phosphatidylinositol; GM3: GM3 ganglioside; PC(O): Alkylphosphatidylcholine; SM: Sphingomyelin; TG [NL]: Triacylglycerol (neutral loss, for associations)

The SNPs used to construct total AC, CE, SM, PI, GM3, PC(O), and TG for BMD/fracture in MR analyses were shown in Supplementary file 2 (Table S4-S11). In the IVW analyses, genetically elevated levels of CE, SM, and PI classes were associated with a lower BMD, while genetically elevated levels of AC class were associated with a higher BMD (Fig. 2A). Genetically elevated levels of GM3, PC(O), and SM were associated with a higher risk of fracture, while genetically elevated levels of TG were associated with a lower risk of fracture (Fig. 2B).

### MR analyses of lipid species for BMD and fracture

**Fig. 3:**
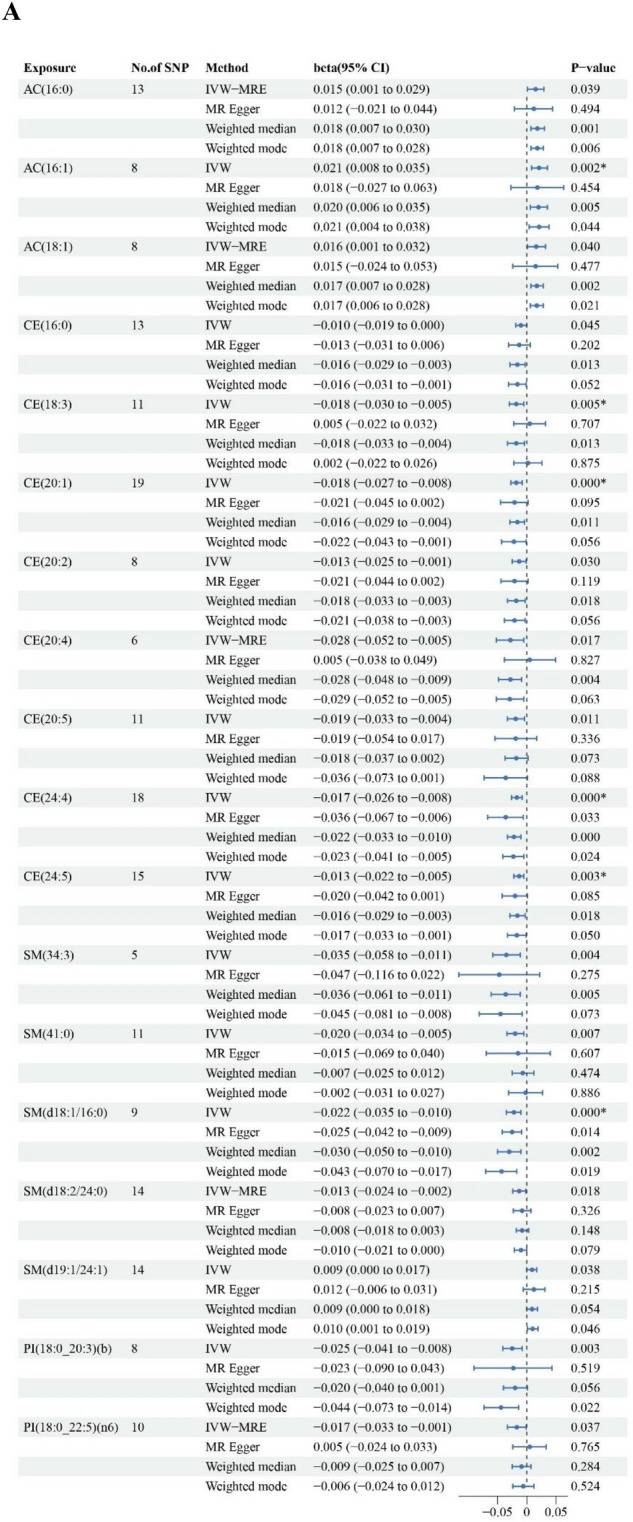

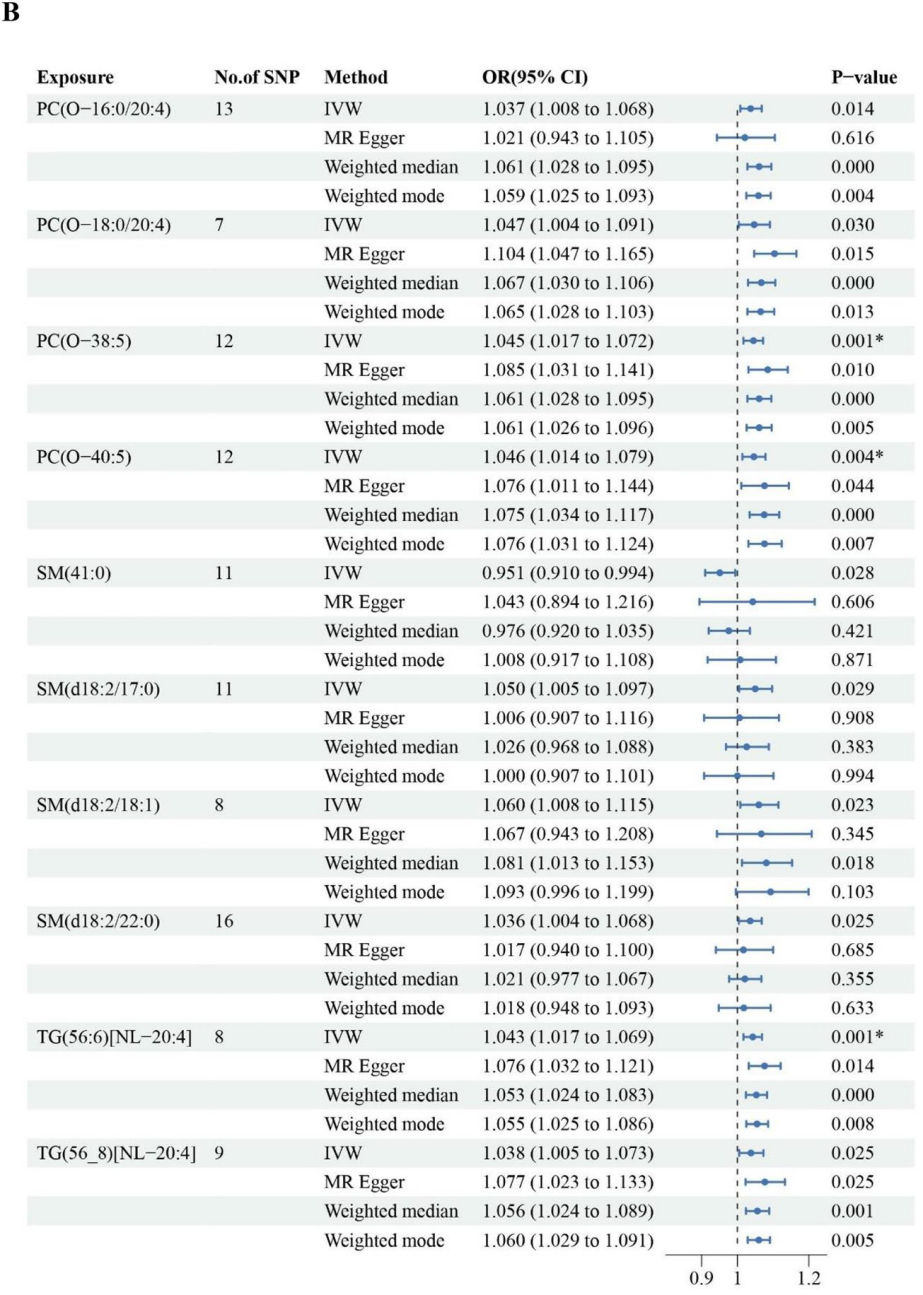
MR analyses at lipid species-level. **A** Forest plot of MR estimates for associations between lipid species and bone mineral density. **B** Forest plot of MR estimates for associations between lipid species and fracture risk. SNP: Single Nucleotide Polymorphism; CI: Confidence Interval; OR: Odds Ratio; IVW: Inverse-Variance Weighted; AC: Acylcarnitine; SM: Sphingomyelin; CE: Cholesteryl ester; PI: Phosphatidylinositol; GM3: GM3 ganglioside; PC(O): Alkylphosphatidylcholine; SM: Sphingomyelin; TG [NL]: Triacylglycerol (neutral loss, for associations)

The SNPs used to construct lipid species of AC, CE, SM, PI, GM3, PC(O), and TG for BMD/fracture in MR analyses were shown in Supplementary file 2 (Table S12-S19). In the IVW analyses on BMD, 18 lipid species were causally associated with BMD (Fig. 3A). Genetically elevated levels of CE(16:0), CE(18:3), CE(20:1), CE(20:2), CE(20:4), CE(20:5), CE(24:4), CE(24:5), SM(34:3), SM(41:0), SM(d18:1/16:0), SM(d18:2/24:0) and PI(18:0/20:3(b)), PI(18:0/22:5(n6)) were associated with a lower BMD, while genetically elevated levels of AC(16:0), AC(16:1), AC(18:1), and SM(d19:1/24:1) were associated with a higher BMD. Of these lipid species, IVW estimates for AC(16:1), CE(18:3), CE(20:1), CE(24:4), CE(24:5) and SM(d18:1/16:0) passed FDR multiple testing.

The associations between lipid species and risk of fracture are shown in Fig. 3B. Ten lipid species were causally associated with fracture risk. Genetically elevated levels of PC(O-16:0/20:4), PC(O-18:0/20:4), PC(O-38:5), PC(O-40:5), SM(d18:2/18:1), SM(d18:2/22:0), SM(d18:2/17:0) and TG(56:6) [NL-20:4], TG(56:8) [NL-20:4] were associated with a higher risk of fracture. Conversely, genetically elevated levels of SM(41:0) were associated with a lower risk of fracture. Of these lipid species, IVW estimates for PC(O-38:5), PC(O-40:5) and TG(56:6) [NL-20:4] passed FDR multiple testing. There were no associations between genetically predicted levels of GM3 species and fracture risk.

### Sensitivity analyses

The MR-Egger, weighted mode, simple mode, and weighted median methods provided similar causal estimates for magnitude (Figs. 2, 3). No evidence of horizontal pleiotropy for lipid classes and species in BMD/fracture was observed (p > 5 × 10^–2^), as determined by the MR-Egger regression intercept approach. Cochrane Q statistics results showed no significant heterogeneity (p > 5 × 10^–2^), except for total PI, AC(16:0), AC(18:1), CE(20:4), PI(18:0_22:5)(n6), SM(d18:2/24:0) in BMD (Supplementary file 2, Table S2-S3). The number of distributions in MR-PRESSO analysis was set to 3000. MR-PRESSO analysis revealed no outliers in the results (global test p > 5 × 10^–2^). Furthermore, no horizontal pleiotropy was found based on the results of the MR-PRESSO and MR-Egger regression intercept analysis. All MR results are presented in Supplementary file 2 (Table S20-S29) in supporting information.

### Biomarker validation

#### Participants characteristics at phase 2

The characteristics of participants at phase 2 who had data on lipids measurement, BMD/fracture and covariates are presented in Table 1. The average age was 64 with 50% being female. At the 2.6-year visit (phase 2), 49% of participants reported a fracture, and at the 10.7-year visit (phase 4), 56% had a fracture.

**Table 1:** Characteristics of participants in the validation cohort*

Characteristics	Participants (n = 492)
Age (years)	64.0 ± 6.4
Female, n (%)	251 (51)
BMI (kg/m^2^)	27.6 ± 4.6
Physical activity (steps per day)	7916.7 ± 3271.7
Current smoking status, n (%)	43 (9)
Serum levels of vitamin D (nmol/L)	60.2 ± 22.2
Falls risk score (z score)	− 0.04 ± 0.75
Use of statins, n (%)	103 (21)
Dietary inflammatory index	− 0.75 ± 1.38
Total hip BMD at phase 2 (g/cm^2^)	0.97 ± 0.14
Total hip BMD at phase 4 (g/cm^2^) (n = 310)	0.95 ± 0.15
Fracture at phase 2, n (%)	241 (49)
Fracture at phase 4, n (%) (n = 310)	174 (56)

^*^Values are the mean ± SD except for percentages. Characteristics of participants were from phase 2 except for hip BMD and fracture

BMI: body mass index, BMD: bone mineral density

#### Associations between lipids and hip BMD

Table 2 shows the associations between the four lipid classes and 18 species and hip BMD over 8 years. AC and PI classes were found to be associated with hip BMD, while SM class was associated with changes in hip BMD. CE(24:5) species were associated with hip BMD, whereas three lipid species (CE(18:3), SM(d18:1/16:0) and SM(d18:2/24:0)) were associated with changes in hip BMD.

**Table 2:** Mixed-effects model of associations between lipids and hip BMD over 8 years

	Hip BMD*		Interaction term of lipids and follow-up time*	
	β (95% CI)	p-value	β (95% CI)	p-value
Lipid classes (per log µM)				
AC	**− 0.013 (− 0.020, − 0.007)**	**8.10E-05**	0.004 (**− **0.006, 0.014)	4.74E-01
CE	**− **0.012 (**− **0.027, 0.003)	1.24E-01	**− **0.012 (**− **0.024, 0.001)	7.51E-02
SM	**− **0.044 (**− **0.14, 0.05)	3.51E-01	**− 0.030 (− 0.049, − 0.011)**	**2.03E-03**
PI	**0.023 (0.021, 0.026)**	**1.96E-07**	**− **0.003 (**− **0.017, 0.010)	6.25E-01
Lipid species (per log µM)				
AC (16:0)	0.004 (**− **0.020, 0.028)	7.64E-01	0.0001 (**− **0.0037, 0.0039)	9.50E-01
AC (16:1)	**− **0.019 (**− **0.037, 0.0001)	5.17E-02	0.008 (**− **0.009, 0.011)	8.75E-01
AC (18:1)	**− **0.013 (**− **0.032, 0.006)	1.64E-01	0.0011 (**− **0.0021, 0.0043)	5.11E-01
CE (16:0)	0.002 (**− **0.014, 0.017)	8.12E-01	**− **0.0025 (**− **0.0060, 0.0011)	1.77E-01
CE (18:3)	**− **0.007 (**− **0.036, 0.022)	6.52E-01	**− 0.012 (− 0.020, − 0.003)**	**9.61E-03**
CE (20:1)	**− **0.009 (**− **0.028, 0.010)	3.54E-01	**− **0.004 (**− **0.014, 0.007)	4.73E-01
CE (20:2)	0.001 (**− **0.011, 0.013)	8.13E-01	**− **0.0020 (**− **0.0048, 0.0008)	1.61E-01
CE (20:4)	**− **0.008 (**− **0.021, 0.005)	2.22E-01	**− **0.0012 (**− **0.0032, 0.0008)	2.36E-01
CE (20:5)	0.003 (**− **0.005, 0.011)	4.59E-01	**− **0.0008 (**− **0.0022, 0.0006)	2.46E-01
CE (24:4)	**− **0.008 (**− **0.022, 0.006)	2.73E-01	**− **0.003 (**− **0.014, 0.006)	4.75E-01
CE (24:5)	**− 0.014 (− 0.020, − 0.009)**	**5.81E-07**	0.002 (**− **0.005, 0.009)	5.15E-01
SM (34:3)	**− **0.004 (**− **0.033, 0.025)	7.86E-01	**− **0.0034 (**− **0.0070, 0.0003)	7.25E-02
SM (41:0)	0.007 (**− **0.013, 0.026)	5.03E-01	**− **0.0021 (**− **0.0053, 0.0010)	1.83E-01
SM (d18:2/24:0)	**− **0.006 (**− **0.032, 0.021)	6.80E-01	**− 0.0042 (− 0.0075, − 0.0010)**	**1.13E-02**
SM (d18:1/16:0)	**− **0.047 (**− **0.150, 0.056)	3.71E-01	**− 0.036 (− 0.058, − 0.015)**	**9.28E-04**
SM (d19:1/24:1)	**− **0.003 (**− **0.025, 0.018)	7.69E-01	**− **0.00004 (**− **0.00304, 0.00297)	9.81E-01
PI (18:0_20:3) (b)	**− **0.005 (**− **0.018, 0.008)	4.58E-01	0.0001 (**− **0.0019, 0.0020)	9.47E-01
PI (18:0_22:5) (n6)	0.010 (**− **0.005, 0.025)	2.12E-01	**− **0.0020 (**− **0.0042, 0.0003)	8.90E-02

Hip BMD: bone mineral density at total hip, AC: Acylcarnitine, SM: Sphingomyelin, CE: Cholesteryl ester, PI: Phosphatidylinositol

^*^Adjusted for age, sex, body mass index, physical activity, current smoking status, serum levels of vitamin d at phase 2. Bold denotes significant associations

The associations between total SM, SM(d18:1/16:0), SM(d18:2/24:0), CE(18:3), CE(24:5), and hip BMD, and changes in hip BMD consistently aligned with directions with the results from IVW analyses.

#### Associations between lipids and fracture risk

Table 3 shows the associations between the four lipids classes and 10 species and the risk of fracture over 8 years. Total PC(O) and TG were found to be associated with a higher risk of fracture, while total GM3 and SM were associated with an increased risk of fracture as the 8-year follow-up period progressed. PC(O-38:5) species were associated with fracture, whereas three lipid species (i.e., SM(d18:2/18:1), SM(d18:2/22:0), SM(d18:2/17:0)) were associated with a progressively increasing risk of fracture over the 8-year follow-up period.

**Table 3:** Mixed-effects model of associations between lipids and fracture risk over 8 years

	Fracture*		Interaction term of lipids and follow-up time*	
	RR (95%CI)	p-value	RR (95%CI)	p-value
Lipid class (per log µM)				
GM3	1.243 (0.855, 1.804)	2.53E-01	**1.235 (1.0001, 1.525)**	**4.99E-02**
PC(O)	**1.226 (1.094, 1.374)**	**1.87E-03**	0.988 (0.770, 1.267)	9.23E-01
SM	1.032 (0.717, 1.464)	8.72E-01	**1.290 (1.025, 1.624)**	**2.97E-02**
TG	**1.006 (1.001, 1.010)**	**1.14E-02**	0.993 (0.912, 1.080)	8.65E-01
Lipid species (per log µM)				
PC (O-16:0/20:4)	1.258 (0.953, 1.662)	1.05E-01	0.986 (0.942, 1.031)	5.25E-01
PC (O-18:0/20:4)	1.081 (0.824, 1.418)	5.75E-01	0.992 (0.953, 1.032)	6.84E-01
PC(O-38:5)	**1.285 (1.092, 1.511)**	**7.32E-04**	0.947 (0.760, 1.178)	6.23E-01
PC(O-40:5)	1.238 (0.911, 1.698)	1.66E-01	1.006 (0.836, 1.209)	9.53E-01
SM (d18:2/18:1)	1.000 (0.771, 1.298)	1.00E + 00	**1.039 (1.003, 1.078)**	**3.51E-02**
SM (d18:2/22:0)	0.788 (0.602, 1.030)	8.15E-02	**1.038 (1.002, 1.075)**	**4.01E-02**
SM (41:0)	1.021 (0.815, 1.280)	8.54E-01	1.019 (0.984, 1.056)	2.85E-01
SM (d18:2/17:0)	0.911 (0.703, 1.180)	4.79E-01	**1.041 (1.003, 1.080)**	**3.30E-02**
TG (56:6)[NL-20:4]	1.160 (0.995, 1.350)	5.75E-02	1.000 (0.911, 1.097)	9.39E-01
TG (56:8)[NL-20:4]	1.051 (0.936, 1.180)	4.00E-01	0.999 (0.981, 1.018)	9.35E-01

^*^Adjusted for age, sex, body mass index, physical activity, current smoking status, falls risk score, and hip BMD at phase 2 and phase 4 as time-dependent variable

GM3: GM3 ganglioside, PC(O): Alkylphosphatidylcholine, SM: Sphingomyelin, TG [NL]: Triacylglycerol (neutral loss, for associations)

Bold denotes significant associations

The directions of the associations between total GM3, PC(O) and SM, PC(O-38:5), SM(d18:2/18:1), SM(d18:2/22:0), SM(d18:2/17:0) and fracture risks were consistent with the results of IVW analyses.

Sensitivity analyses adjusting for statin use and dietary inflammatory index showed that the associations between lipid species and both BMD and fracture risk remained consistent with our primary findings (Supplementary Tables S30-S31). Additionally, the results from the inverse probability weighting-adjusted models were consistent with the main findings (data not shown), indicating that loss to follow-up did not materially affect the associations observed.

## Discussion

In this study, we integrated two-sample MR with observational analyses to reveal novel associations of lipids with changes in BMD and fracture risk. Our two-sample MR analyses identified four lipid classes, encompassing 18 species, causally linked to BMD. Further leveraging data from a population-based prospective cohort, we observed genetically elevated levels of three lipid classes and four distinct species correlated with hip BMD or its changes. Additionally, we identified and validated the involvement of four lipid classes and their species in elevating fracture risk over 8 years, and these relationships were independent of covariates as well as hip BMD changes and falls risk score. These findings, obtained from both genetic and observational analyses, suggest that alterations in lipid metabolism play a crucial role in bone remodeling and fracture risk.

The current study found genetically elevated levels of SM associated with both BMD and fracture risk in the MR analyses. Further, our observational findings revealed that SM was associated with changes in hip BMD, as well as a greater risk of fracture over 8 years. SM, as a crucial component of cell membranes, has been shown to be involved in cell survival, proliferation, migration, and inflammation processes that are inherently linked to bone health [32]. The breakdown of SM into phosphocholine and ceramide, both vital for normal bone mineralisation [33], highlights a direct impact of SM metabolism on bone structure and density. Beyond the direct impact on bone mineralisation, the involvement of SM in inflammatory and immune responses may explain its association with fracture risk [34]. Previous genetic studies have identified variants in the sphingomyelin synthase gene (*SGMS1*) that are linked to osteoporosis. Furthermore, presence of specific gene mutation in *SGMS1* has been linked to early onset osteoporosis and cranial sclerosis, providing a genetic backdrop to our findings [35]. These genetic variations in the SM synthesis pathway suggest the possibility of a genetically predisposed influence of SM on bone health. In the current study, SM species linked to changes in hip BMD differed from those related to fracture risk. These variations may reflect distinct functionality of SM species and their multifaceted role in bone health [36].

In this study, MR analysis also identified causal links between classes of AC, CE, and PI and their species with BMD. Validation in the longitudinal cohort confirmed the associations of AC and PI with hip BMD. Specifically, CE(24:5) was found to be connected to hip BMD, and CE(18:3) was associated with BMD changes. Our findings on AC align with and expand upon one previous study that identified a causal connection between very short chain AC (acetylcarnitine and propionylcarnitine) and heel BMD [37]. AC plays a key role in transporting fatty acids into mitochondria for β-oxidation, where they are converted into energy [38, 39]. This process supplies 40% to 80% of the energy needs of osteoclasts, highlighting a vital role of fatty acid oxidation in bone cell energy metabolism [40]. PI species might contribute to chronic inflammatory processes, potentially leading to bone loss in osteoporosis. The metabolism of PI plays a role in the signaling mechanism of the receptor activator of nuclear factor κB (RANK), which is a key local factor in regulating osteoclastogenesis and bone resorption [41]. Although no link was found between CE and BMD in the validation cohort, one CE species (CE(18:3)) was associated with changes in BMD. This association, consistent with our MR analysis, suggests that certain CE species may play a role in bone remodeling. The presence of CE(18:3), an omega-3 fatty acid, may imply that dietary intake of omega-3 s or their metabolic conversion to longer-chain omega-3 s may influence BMD.

The associations of lipid classes of GM3, PC(O), and TG and three species with fracture were identified in MR analyses. Additionally, higher levels of GM3 were found to be associated with a greater fracture risk over 8 years, which showed consistency in direction with the MR results. GM3, a crucial glycosphingolipid, plays a role in modulating immune responses and inhibiting fibroblast growth factor receptors, potentially linking it to bone resorption and osteoporosis through its effects on inflammation [42]. In our validation cohort, the associations between PC(O) and PC(O-38:5) with fracture were consistent with their effect sizes and directions observed in MR analyses, suggesting a causal role of these lipids. These consistencies indicate that PC(O) and PC(O-38:5) likely have an important role in the biological processes associated with fracture. PC(O) may also influence fracture healing through its critical roles in maintaining cell membrane integrity, modulating signaling pathways relevant to bone metabolism, regulating inflammatory responses at the fracture site, and affecting cell survival and apoptosis [43].

The current study failed to validate TGs in association with fracture risk in the validation cohort, albeit a causal link observed in MR analysis. There is evidence that circulating TGs are subject to metabolic and lifestyle controls, it is possible that these factors could confound the validation analysis. Bone formation is a highly energy-demanding process that can be influenced by metabolic disorders [44]. Reduced TG metabolism has been found in osteoporosis bone tissues, characterized by decreased osteoblastogenesis and increased osteoclastogenesis [45]. Loss of function of an essential lipase of TG hydrolysis, lysosomal acid lipase (*LAL*), has been found to profoundly impact osteoblastogenesis and increase fracture risk [46]. A possible explanation may be due to the dual effects of TG metabolism in bone remodeling. As fatty acids derived from TG metabolism produce more energy, the effect of fatty acids on the bioactive process of osteoblast is still controversial [47].

This study has several strengths. First, we identified several novel causal associations between lipid classes and species with BMD changes and fractures. Although many of the identified lipid species are ubiquitous and involved in systemic metabolic processes, their consistent associations with bone outcomes suggest that they may play a relevant, though not necessarily exclusive, role in skeletal metabolism. Several of the lipid species identified such as CE and AC are influenced by diet, metabolic status, and lipid-lowering medications, suggesting that dietary modification or pharmacological intervention may hold potential for improving bone health. Second, by employing two-sample MR analysis, we minimized observational biases, such as confounding factors and reverse causality. Third, MR findings were validated in an independent cohort, further enhancing the robustness and reliability of our findings. However, minor limitations of the current study should be noted. First, to avoid the bias of population stratification, the summary-level data of the previous GWAS only included European ancestries, which may limit generalizability to other ancestries. The BMD trait was measured in the heel in the UK Biobank, rather than the femoral neck BMD used in Genetics Factors for Osteoporosis (GEFOS), as GEFOS included a mixed population. Second, due to the small number of incident fractures, particularly for hip and vertebral fractures, we were unable to stratify analyses by fracture site, which may have obscured site-specific associations with differing prognostic relevance. Lastly, the fractures of participants relied on self-reporting, which may lead to an overestimation or underestimation of the relationship between lipids and fracture risk [48]. However, the effect sizes and directions of lipids in observational analyses were similar to those observed in MR analyses.

In conclusion, these findings suggest that alterations in lipid metabolism play a role in bone remodeling and fracture risk, and support investigating lipids for hip BMD change and identifying patients at ‘high risk’ of osteoporotic fracture.

## Supplementary Information

Below is the link to the electronic supplementary material.

Supplementary file1 (DOCX 27 KB)

Supplementary file2 (XLSX 297 KB)

## Data Availability

The data that support the findings of this study are available from the corresponding author upon reasonable request.

## Declarations

### Graphical abstract

This study explores the causal relationship between lipid metabolism and changes in bone mineral density (BMD) and fracture risk through integrated two-sample Mendelian randomization (MR) and observational analysis. The two-sample MR analysis uncovers causal relationships between lipid classes and species with BMD and fractures. Validation in an independent cohort confirms associations for specific lipid species with hip BMD change and fracture risk over an 8-year period. These findings suggest lipid metabolism influences bone remodeling and fracture risk, highlighting the potential for targeted interventions and risk assessment in fracture prevention.
