# Supplementary material for: Identification and Validation of Novel Lipids Linked to Bone Mineral Density Change and Fracture Risk
Source: Calcif Tissue Int. 2025 Jun 25;116(1):89. doi: 10.1007/s00223-025-01399-1 (PMC12198305; doi:10.1007/s00223-025-01399-1)
Supplement: Supplementary file 1 — Supplementary file1 (DOCX 27 KB) [file 223_2025_1399_MOESM1_ESM.docx]

**Supplementary texts**

**Lipidomic analysis**

Lipidomic analysis was conducted using an Agilent 6495C triple quadrupole liquid chromatography/tandem mass spectrometry (LC/MS/MS) in conjunction with an Agilent 1290 series HPLC system and a ZORBAX eclipse plus C18 column (Agilent, Santa Clara, CA, USA) operating in positive/negative switching polarity, as detailed previously (1, 2). To ensure the integrity of the analysis, technical quality controls (TQCs) and pooled serum QCs (PQCs) were implemented to monitor the overall performance of LC/MS/MS analysis and address experimental issues arising during lipid extraction. NIST1950 samples, which represent the “population” lipidomic profile, were utilised as a reference material to facilitate future alignment with other studies. Chromatographic peaks corresponding to each lipid were integrated utilising the MassHunter software (Agilent). Subsequently, the peak areas were utilised to determine the concentrations of lipid species by comparing them with the relevant internal standard. Mean and median coefficients of variation (CVs) of all TQCs were ≤11.7%, and those for PQCs were 9.4% and 7.6%, respectively (3).

**Cochran’s Q statistic, MR-PRESSO analysis, F-statistic and Steiger filtering method**

Cochran’s Q statistic was employed for IVW analysis, while the Rucker’s Q statistic was utilised for MR-Egger analysis to detect the heterogeneity of the effects of SNPs related to lipids on BMD/fracture outcomes. A p-value greater than 5×10^-2^ indicates no heterogeneity (4). The MR-PRESSO analysis detects and attempts to reduce horizontal pleiotropy by removing significant outliers. The MR-PRESSO outlier test relies on InSIDE assumptions and requires that at least 50% of the genetic variants are valid instruments (5). MR-Egger regression was employed to identify potential pleiotropy and assess the impact of pleiotropy on the risk estimation of the intercept test. A p-value greater than 5×10^-2^ indicates no pleiotropy (6). The strength of IVs was evaluated by calculating the F-statistic (7). A F-value less than 10 indicated a weak instrument, which were therefore excluded (8). Additionally, the Steiger filtering method was applied to ensure the directionality of the association between lipids and BMD/fractures. Results are presented as a categorical variable: ‘true’ if the effect direction is from exposure to outcome at p<5×10^-2^; ‘false’ if reversed at p<5×10^-2^; and ‘uncertain’ if p≥5×10^-2^ (9).

**Reference**

1. Beyene HB, Olshansky G, AA TS, Giles C, Huynh K, Cinel M, et al. High-coverage plasma lipidomics reveals novel sex-specific lipidomic fingerprints of age and BMI: Evidence from two large population cohort studies. PLoS Biol. 2020;18(9):e3000870.

2. Huynh K, Mellett N, Duong T, Nguyen A, Meikle T, Giles C, et al. A Comprehensive, Curated, High-Throughput Method for the Detailed Analysis of the Plasma Lipidome. 2021.

3. Ma C, Liu M, Tian J, Zhai G, Cicuttini F, Schooneveldt YL, et al. Lipidomic Profiling Identifies Serum Lipids Associated with Persistent Multisite Musculoskeletal Pain. Metabolites. 2022;12(3).

4. Greco MF, Minelli C, Sheehan NA, Thompson JR. Detecting pleiotropy in Mendelian randomisation studies with summary data and a continuous outcome. Statistics in medicine. 2015;34(21):2926-40.

5. Verbanck M, Chen CY, Neale B, Do R. Detection of widespread horizontal pleiotropy in causal relationships inferred from Mendelian randomization between complex traits and diseases. Nature genetics. 2018;50(5):693-8.

6. Bowden J, Davey Smith G, Burgess S. Mendelian randomization with invalid instruments: effect estimation and bias detection through Egger regression. International journal of epidemiology. 2015;44(2):512-25.

7. Gill D, Efstathiadou A, Cawood K, Tzoulaki I, Dehghan A. Education protects against coronary heart disease and stroke independently of cognitive function: evidence from Mendelian randomization. International journal of epidemiology. 2019;48(5):1468-77.

8. Burgess S, Thompson SG. Bias in causal estimates from Mendelian randomization studies with weak instruments. Statistics in medicine. 2011;30(11):1312-23.

9. Deng Y-T, Ou Y-N, Wu B-S, Yang Y-X, Jiang Y, Huang Y-Y, et al. Identifying causal genes for depression via integration of the proteome and transcriptome from brain and blood. Molecular Psychiatry. 2022;27(6):2849-57.
